# Carbon-ion radiotherapy subsequent to balloon-occluded retrograde transvenous obliteration for hepatocellular carcinoma with hepatic encephalopathy: a multidisciplinary approach

**DOI:** 10.1007/s12328-021-01395-6

**Published:** 2021-04-03

**Authors:** Naoto Osu, Shintaro Shiba, Kei Shibuya, Shohei Okazaki, Yuhei Miyasaka, Masahiko Okamoto, Tatsuya Ohno

**Affiliations:** 1 Department of Radiation Oncology, Gunma University Graduate School of Medicine, 3-39-22, Showa-machi, Maebashi, Gunma 371-8511 Japan; 2 Gunma University Heavy Ion Medical Center, 3-39-22, Showa-machi, Maebashi, Gunma 371-8511 Japan

**Keywords:** Hepatocellular carcinoma, Hepatic encephalopathy, Carbon-ion radiotherapy, Balloon-occluded retrograde transvenous obliteration

## Abstract

Radical treatments of hepatocellular carcinoma (HCC) with hepatic encephalopathy (HE) can be often difficult due to poor liver function or disturbance of consciousness. An effective treatment requires a combinatorial approach incorporating a treatment for HE and radical therapy for HCC that does not compromise liver function. Here, we report a case of a 78-year-old Japanese male with HCC and HE caused by splenorenal shunt. Serum ammonia levels were high. He was not suitable for surgery, percutaneous radiofrequency ablation, or transarterial chemoembolization due to the location of the tumor and poor liver function, which included HE. Thus, he underwent BRTO, with an immediate improvement in both HE and serum ammonia levels. After BRTO, he received C-ion RT as a radical treatment for HCC. After treatment, HCC was well controlled; however, at 35 months post-initiation of C-ion RT, he developed local recurrence without a further reduction in liver function status. Therefore, we repeated C-ion RT. The patient remains alive at 3 months post-treatment, with no evidence of local recurrence, distant metastasis, or toxicity. Although this is a single case report, it suggests that a combinatorial treatment consisting of BRTO and C-ion RT may increase survival rates of patients with HCC and HE.

## Introduction

Liver function is one of the most important prognostic factors with respect to treatment of hepatocellular carcinoma (HCC); indeed, patients with poor liver function are more difficult to cure [1, 2]. Although hepatic resection is the first choice treatment for HCC, patients with poor liver function (i.e., the Barcelona Clinic Liver Cancer [BCLC] classification stage B and C), and/or hepatic encephalopathy (HE), and/or portal hypertension are contraindicated [1, 2]. Percutaneous radiofrequency ablation (RFA) and transarterial chemoembolization (TACE) are viable treatment options for inoperable patients (i.e., those with BCLC stage 0, A, B, and those that refuse surgery). However, these treatments have limitations. It is difficult to apply RFA to large tumors, lesions adjacent to the large vessels, lesions that are undetectable by ultrasound, and in cases where portal blood flow is reduced by a portosystemic shunt; such patients are at high risk of liver-related complications and exacerbation of HE after TACE.

Use of carbon-ion radiotherapy (C-ion RT) to treat HCC has shown favorable clinical results, with minimal liver damage [3–6]. These favorable results are due to the biological and physical advantages that C-ion RT has over X-ray RT [7–9]. However, C-ion RT for HCC with HE presents technical difficulties in patients who cannot maintain a static position during irradiation due to flapping tremor or disturbance of consciousness. Therefore, it is necessary to control HE before C-ion RT. In this respect, balloon-occluded retrograde transvenous obliteration (BRTO) is considered an effective treatment.

Here, we report a patient with HCC with a favorable clinical course who received sequential treatment with BRTO and C-ion RT. For this patient, standard therapies were not considered applicable.

## Case report

**Fig. 1:**
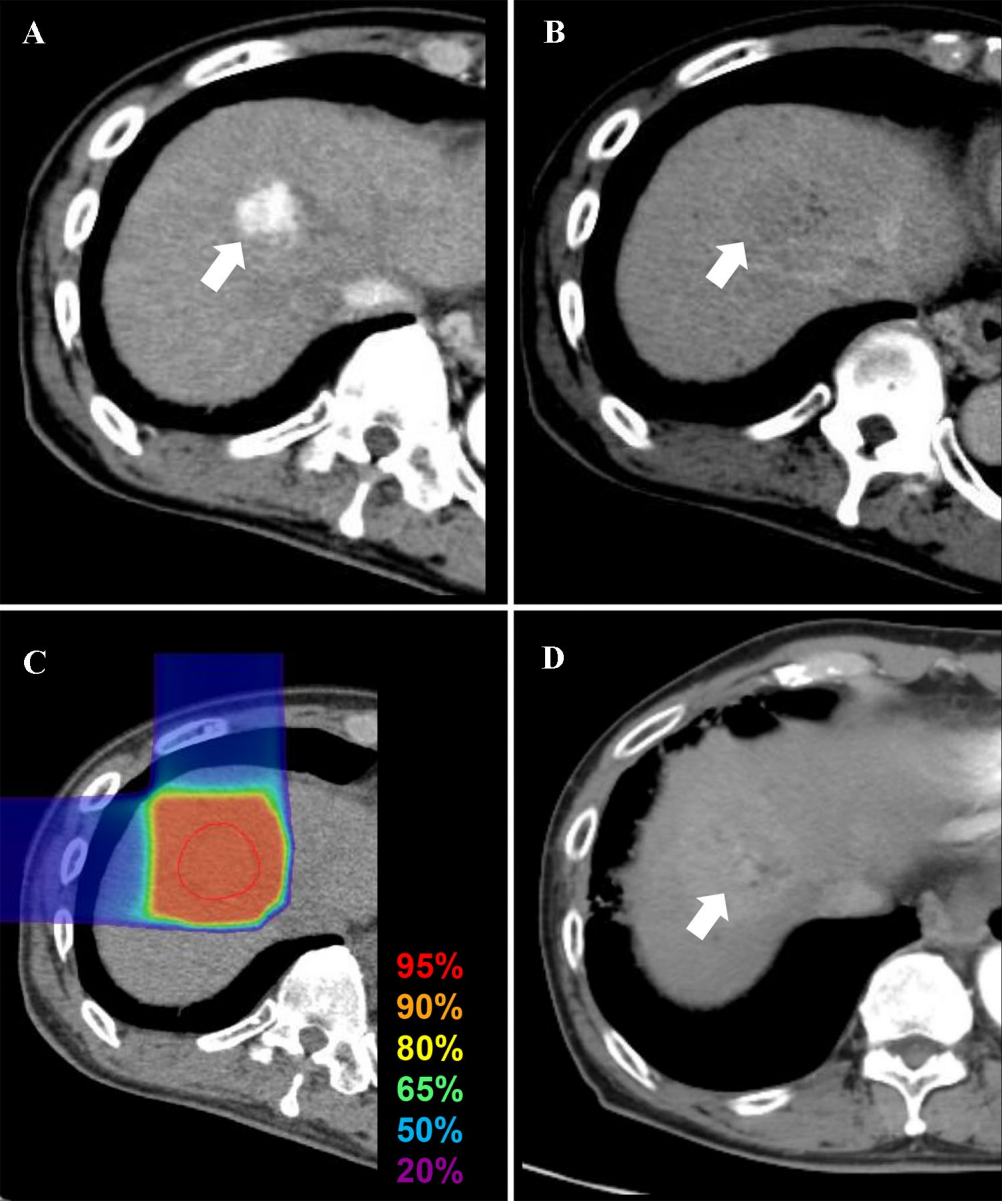
Contrast-enhanced computed tomography (CT) and dose distribution of carbon-ion radiotherapy (C-ion RT) for hepatocellular carcinoma (HCC). **a** Axial CT image taken in the arterial phase before C-ion RT. The white arrow shows HCC with arterial enhancement. **b** CT image taken in the delayed phase. The white arrow shows slight washout. **c** Dose distribution of C-ion RT. The area within the red outline is the gross tumor volume (GTV). Highlighted are the 95% (red), 90% (orange), 80% (yellow), 65% (green), 50% (blue), and 20% (purple) isodose curves (100% = 60.0 Gy [RBE]). **d** CT image taken in the arterial phase 6 months after primary C-ion RT, showing disappearance of the tumor

**Fig. 2:**
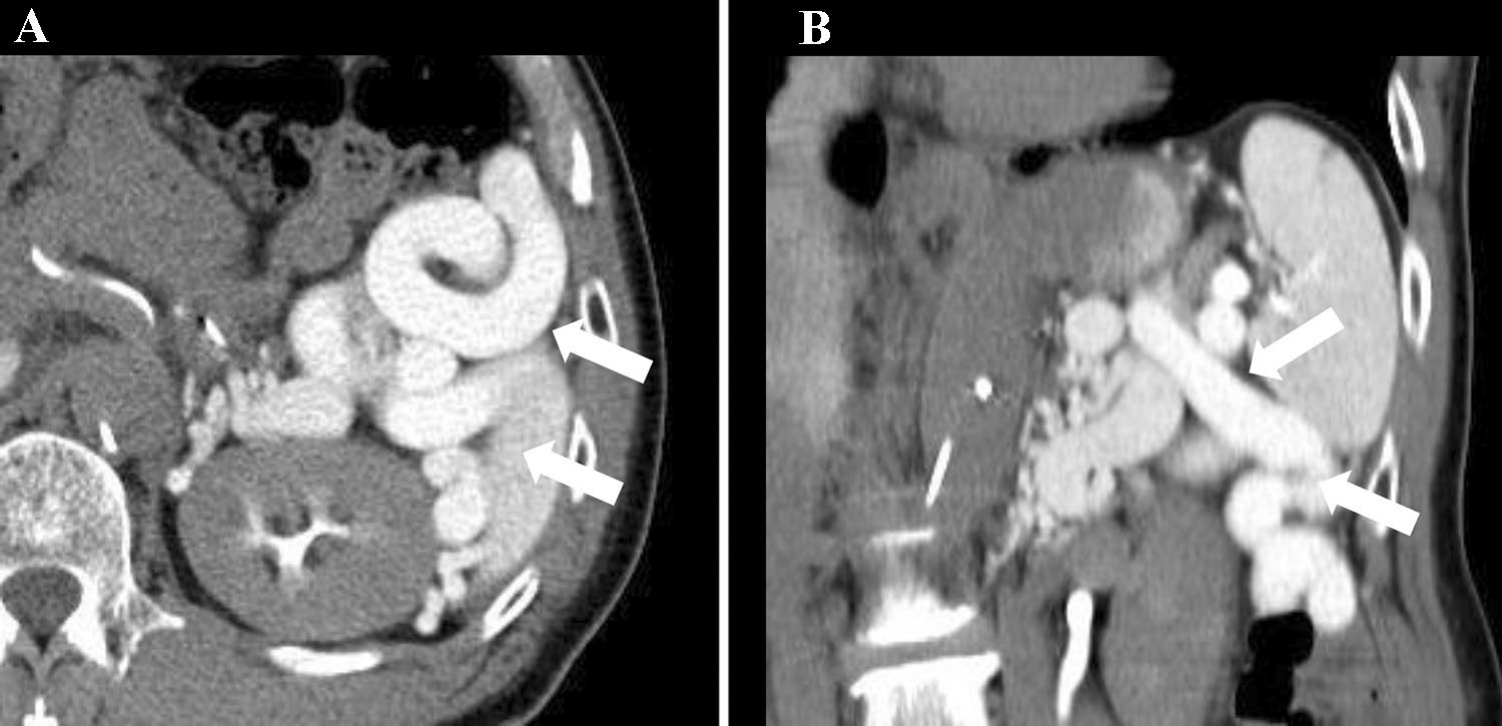
Computed tomography (CT) images showing the splenorenal shunt. **a** Axial CT image. **b** Coronal CT image. The white arrow shows the splenorenal shunt

A 78-year-old Japanese male with HCC was referred to the Gunma University Heavy Ion Medical Center for C-ion RT. He had liver cirrhosis caused by infection with hepatitis C virus (genotype 2a), which was previously treated with interferon/ribavirin, but a sustained virologic response was not achieved. His performance status was 1. Liver function status was Child–Pugh class C (score: 10), with grade III HE (evaluated according to West Haven criteria), BCLC stage C, and modified albumin–bilirubin (mALBI) grade 3 (score: − 1.15) [2, 10]. Laboratory tests showed elevated levels of serum ammonia (241 μg/dL; institution reference range 3–47 μg/dL), alpha-fetoprotein (3.5 IU/L; reference range 0–7.0 IU/L), and des-γ-carboxy prothrombin (DCP) (83 AU/mL; reference range < 40 AU/mL), total bilirubin (1.3 mg/dL; reference range 0.3–1.2 mg/dL), albumin (2.4 mg/dL; reference range 3.9–5.0 mg/dL), prothrombin time (53%; reference range 70–130%), platelets (78,000 cells/μL; reference range 160,000–350,000 cells/μL), HCV-RNA (5.2 log_10_ IU/mL). He was also anti-HCV antibody positive. The indocyanine green retention rate at 15 min (ICG15) was 51.4%. Previously, HE was treated with systemic medication (rifaximin) and branched chain amino acids. Computed tomography (CT) and gadolinium-ethoxybenzyl-diethylenetriamine pentaacetic acid (Gd-EOB-DTPA)-enhanced magnetic resonance imaging (MRI) revealed a nodular tumor (2.6 × 2.5 × 2.1 cm) in segment eight, with early enhancement in the arterial phase and slight washout in the delayed phase (Fig. 1a, b). MRI and CT showed a portosystemic shunt from the splenic vein to the left renal vein (splenorenal shunt) (Fig. 2), no ascites, and no evidence of metastasis to lymph nodes and distant organs. The patient was diagnosed with Stage IB (clinical T1bN0M0) HCC according to the 8th edition of the Union for International Cancer Control/American Joint Committee on Cancer TNM staging system.

**Fig. 3:**
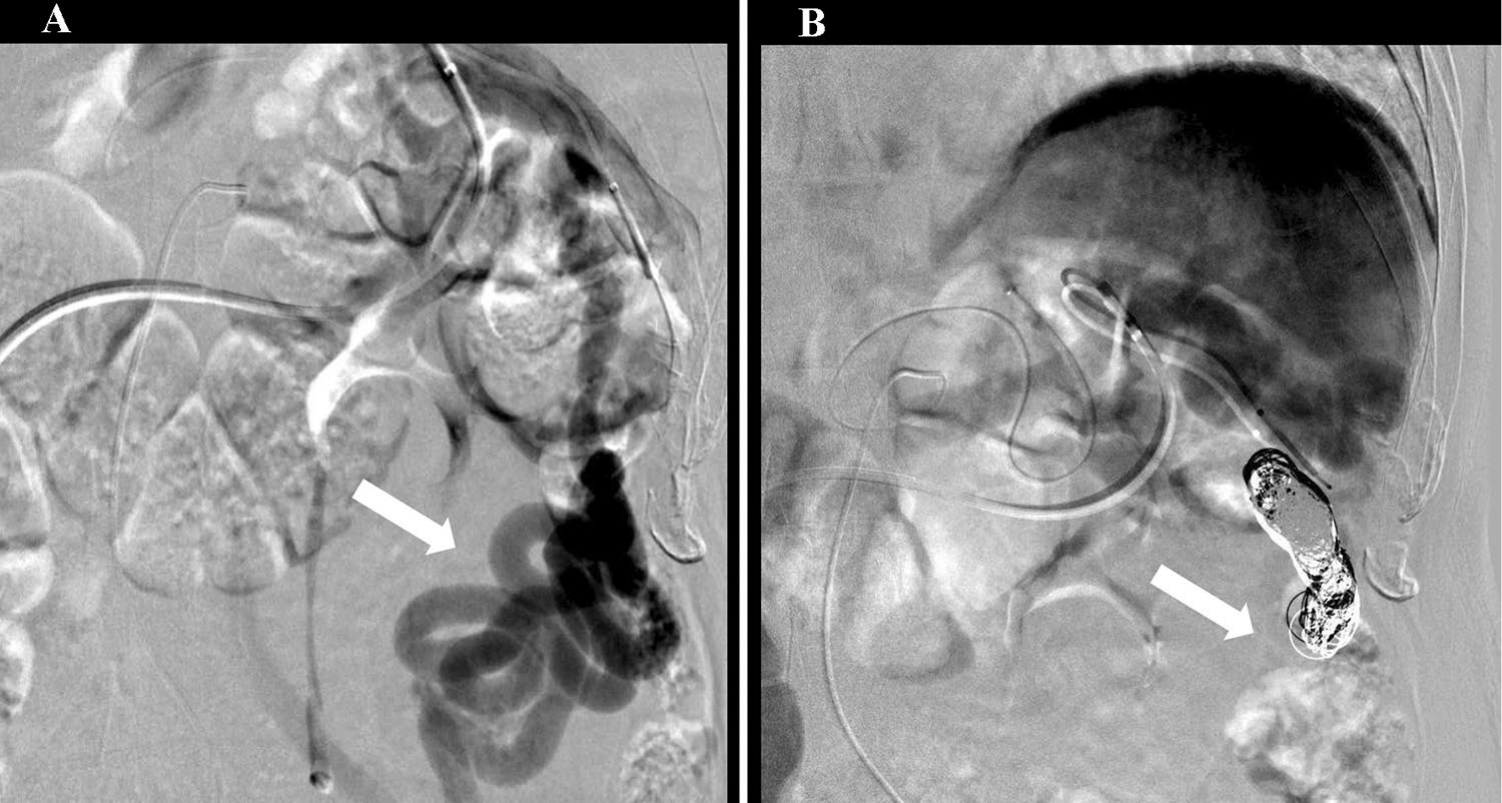
Angiography before and after balloon-occluded retrograde transvenous obliteration (BRTO). **a** Before BRTO. **b** After BRTO, showing disappearance of the shunt flow. The white arrow shows the splenorenal shunt

Surgery, RFA, and TACE were not indicated due to poor liver function (mALBI grade 2b; ICG15, 51.4%), tumor location (adjacent to vascular structures), and portal hypertension with a portosystemic shunt. Therefore, C-ion RT was considered as an alternative treatment option for HCC, however, it was deemed difficult due to impaired consciousness caused by exacerbation of HE. The splenorenal shunt was considered to be the main cause of HE; therefore, the treatment strategy was discussed with the cancer board of our hospital, and C-ion RT was recommended after HE was improved by occluding the splenorenal shunt with BRTO (Fig. 3).

**Fig. 4:**
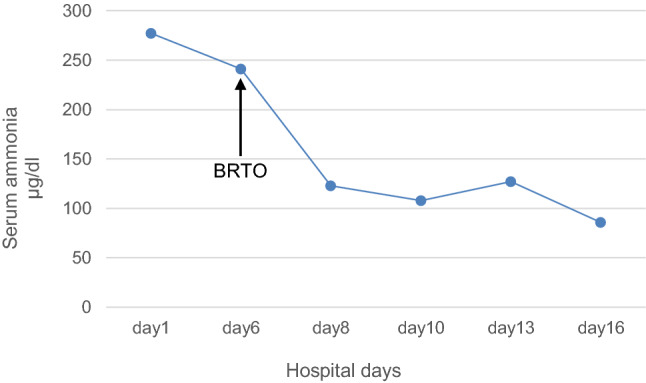
Kinetics of serum ammonia levels before and after balloon-occluded retrograde transvenous obliteration. *BRTO* balloon-occluded retrograde transvenous obliteration

After BRTO, HE, serum ammonia levels, and liver function improved immediately with no exacerbation of ascites (Table 1; Fig. 4). Despite the improvement in liver function, there was no change in the treatment strategy for HCC because poor liver function (mALBI grade, 2b; ICG15, 44.3%) precluded surgery and C-ion RT potentially has a higher local effect than TACE [7]. C-ion RT began 1 month after BRTO. The dose of C-ion RT was 60.0 Gy [Relative Biological Effectiveness (RBE)], delivered in four fractions (Fig. 1c). The patient completed C-ion RT with no acute toxicity. After C-ion RT, there was no evidence of local recurrence, distant metastasis, or toxicity for about 3 years (i.e., no recurrence of HE, no obvious exacerbation of liver function, and no acute exacerbation of HCV infection) (Table 2; Fig. 1d). After the tumor disappeared following C-ion RT, he was treated with a 12-week course of glecaprevir/pibrentasvir (GLE/PIB), which resulted in a sustained virologic response (SVR).

**Table 1:** Parameters of liver function before and after BRTO

	Before BRTO	1 month after BRTO
HE	Grade III	Grade 0
Ascites	None	None
T-bil (mg/dL)	1.3	1.3
Alb (mg/dL)	2.4	2.8
PT ratio (%)	53	77
ICG15 (%)	51.4	44.3
Child–Pugh	C (score: 10)	A (score: 6)
mALBI grade	3 (score: − 1.15)	2b (score: − 1.49)

*HE* hepatic encephalopathy (evaluated by West Haven criteria), *T-bil* total bilirubin (reference range 0.3–1.2), *Alb* albumin (reference range 3.9–5.0), *PT* prothrombin time (reference range 70–130), *ICG15* indocyanine green retention rate at 15 min, *BRTO* balloon-occluded retrograde transvenous obliteration

**Table 2:** Laboratory test data before and after C-ion RT

	Before C-ion RT	3 months	6 months	12 months	15 months	27 months
HE	Grade 0	Grade 0	Grade 0	Grade 0	Grade 0	Grade 0
Ascites	None	None	None	None	None	None
T-bil (mg/dL)	1.3	1.4	2.0	2.7	1.7	1.9
Alb (mg/dL)	3.0	3.1	3.2	2.9	3.1	3.5
PT ratio (%)	76	75	75	74	76	79
Child–Pugh	A (score: 6)	A (score: 6)	B (score: 7)	B (score: 7)	A (score: 6)	A (score: 6)
mALBI grade	2b (score: − 1.66)	2b (score: − 1.72)	2b (score: − 1.71)	3 (score: − 1.37)	2b (score: − 1.67)	2b (score: − 1.98)
HCV-RNA (log_10_ IU/mL)	5.2	5.8	6.1	N.D	N.D	N/A

*C-ion RT* carbon-ion radiotherapy, *N.D.* not detected, *N/A* not applicable

**Fig. 5:**
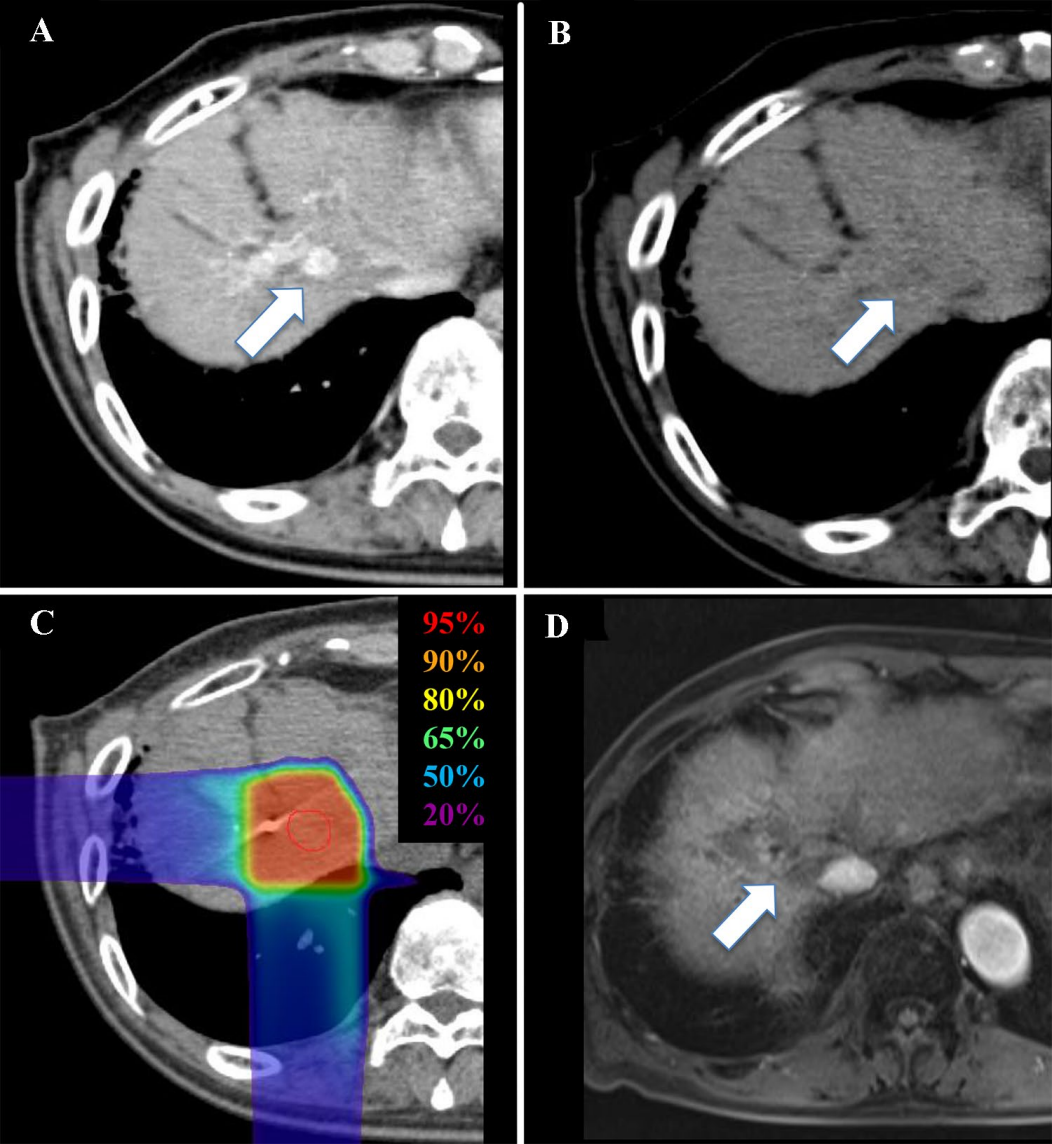
Computed tomography (CT), magnetic resonance imaging (MRI), and dose distribution of carbon-ion radiotherapy (C-ion RT) for local recurrence of hepatocellular carcinoma (HCC). **a** Axial CT image taken in the arterial phase. The white arrow shows recurrence of HCC with arterial enhancement. **b** CT image taken in the delayed phase. The white arrow shows slight washout. **c** Dose distribution of C-ion RT for recurrent HCC. The area within the red outline is the GTV. Highlighted are the 95% (red), 90% (orange), 80% (yellow), 65% (green), 50% (blue), and 20% (purple) isodose curves (100% = 60.0 Gy [RBE]). **d** MRI image taken in the arterial phase three months after re-irradiation with C-ion RT. The white arrow shows tumor shrinkage

However, at 35 months from initiation of C-ion RT, MRI revealed local recurrence for HCC, with no evidence of metastasis to lymph nodes or distant organs (Fig. 5a, b). Liver function status at the time of local recurrence was Child–Pugh class A (score: 5), BCLC stage C, and mALBI grade 2b (score: − 2.25). The ICG15 was 41.6 (%). Therefore, we scheduled and performed re-irradiation with C-ion RT at 60.0 Gy [RBE], delivered in four fractions (Fig. 5c). The patient completed C-ion RT without any acute toxicity. Three months after C-ion RT, CT showed tumor shrinking (Fig. 5d) and there was no evidence of local recurrence, distant metastasis, or recurrence of HE.

## Discussion

Here, we show that BRTO can control HE and enable radical treatment for HCC with C-ion RT. In addition, there was no exacerbation of poor liver function, meaning that C-ion RT could be repeated to treat the recurrent lesion. BRTO and C-ion RT for HCC may prolong survival in this case.

Retrograde transvenous obliteration (RTO), including BRTO, is an established treatment for gastric varices [11, 12]. However, RTO is not an established treatment for HE, although the efficacy and safety of RTO as a treatment for HE have been reported [12–14]. Lee et al. reported 91% clinical cases of HE that were treated successfully by RTO; follow-up was over 2 years, with low rates of tolerable toxicity [14]. In addition, 67% of patients had complete resolution of their HE symptoms during a follow-up period of 893 ± 585 days (range 36–1881 days, median 755.0 days). Ishikawa et al. reported that BRTO significantly improves hyperammonemia and liver function in portal hypertensive patients with refractory HE by increasing portal flow [15]. In fact, there was an improvement in HE and liver function after BRTO in our case. To the best of our knowledge, no report has described use of BRTO for HCC patients with HE prior to radical treatment. Our results show that BRTO is an option that may enable HCC patients with HE to receive radical local treatment.

C-ion RT delivers higher local doses than X-ray RT (i.e., stereotactic body RT and intensity-modulated RT) [8, 9]; also, the modality enables delivery of a reduced dose to the liver while ensuring good target coverage. Therefore, the effect on liver function is minimal, and exacerbation of poor liver function and radiation-induced liver disease after C-ion RT are rare [3–6]. This ability to preserve liver function might contribute to survival [16]. In this case, there was no obvious exacerbation of the Child–Pugh class or mALBI grade after C-ion RT, and preservation of liver function enabled re-irradiation with C-ion RT to treat local recurrence. We believe that radical cancer treatment with C-ion RT, coupled with preservation of liver function, prolonged survival in this case.

## Conclusion

This report suggests that a combinatorial treatment consisting of BRTO and C-ion RT may prolong survival of patients with HCC and HE. This is because combination treatment enables HCC patients with HE to receive radical local treatment.
